# Demographic and clinical correlates of sexual dysfunction among Nigerian male outpatients on conventional antipsychotic medications

**DOI:** 10.1186/1756-0500-5-267

**Published:** 2012-06-07

**Authors:** Aina Kikelomo Oyekanmi, Adegoke Oloruntoba Adelufosi, Olukayode Abayomi, Timothy Olaolu Adebowale

**Affiliations:** 1 Neuropsychiatric Hospital, Aro, P.O Box 2210, Sapon, Abeokuta, Ogun State, Nigeria; 2 Ladoke Akintola University Teaching Hospital, Ogbomoso, Oyo State, Nigeria

**Keywords:** Sexual dysfunction, Conventional antipsychotics, Schizophrenia

## Abstract

**Background:**

In psychotic disorders, early intervention with antipsychotic medications increases the likelihood of favourable long-term course. However, the pharmacologic management especially with conventional antipsychotic medications is complicated by a high rate of adverse effects including sexual dysfunction. This study aims to determine the demographic and clinical factors associated with sexual dysfunction among male psychiatric outpatients on conventional antipsychotic medications in South-western Nigeria.

**Methods:**

Two hundred and seventy five consecutive male outpatients with psychotic disorders on conventional antipsychotic medications were interviewed. Data was collected on demographic characteristics, illness-related and medication-related variables. Illness severity was assessed with the Brief psychiatric rating scale. The International Index of Erectile Function questionnaire was used to assess for sexual dysfunctions.

**Results:**

A total of 111 (40.4%) respondents had one or more forms of sexual dysfunction. Sexual desire dysfunction was present in 47 (17.1%) of respondents, erectile dysfunction in 95 (34.5%), orgasmic dysfunctions in 51 (18.5%), intercourse dissatisfaction in 72 (26.2%) and overall dissatisfaction in 64 (23.3%). Sexual dysfunction was significantly associated with employment status, age, marital status, haloperidol use, medication dosage, and presence of psychopathology. Unemployment was the only significant independent correlate of sexual dysfunction, with unemployed respondents twice more likely to have sexual dysfunction compared with those employed (Wald = 3.865, Odds Ratio = 2.033, 95% confidence interval = 1.002 - 4.124, p = 0.049).

**Conclusions:**

The high prevalence of sexual dysfunction found in this study suggests a need among clinicians for increased awareness and recognition of the sexual side effects in patients taking conventional antipsychotic medications. This knowledge should guide conventional antipsychotic medication prescription in the at-risk population to improve treatment adherence.

## Background

Sexual dysfunction is commonly associated with the pharmacologic management of psychotic illnesses especially conventional antipsychotic medications [1-3]. This is further complicated by the effects of major psychotic illness itself on sexual functioning, among which are reduced libido, decreased sexual performance and satisfaction [4].

Previous studies showed that sexual dysfunction occurred in as many as 60% of outpatients with schizophrenia [5-7]. Sexual adverse effects have been reported in up to 45–50% of patients taking conventional antipsychotics and are more likely to be disturbing to men than women [8-10]. Other authors reported that more than 50% of males on conventional antipsychotics experienced sexual dysfunction [11]. Commonly experienced sexual side effects in males are poor penile erection, ejaculatory and orgasmic disturbance [12,13]. Despite this high rate, complaints about sexual dysfunction are largely unexplored or ignored by clinicians, or attracted only vague reassurances resulting in poor medication adherence and quality of life [2,14,15].

There is a paucity of studies on sexual dysfunction in Nigeria [16]. Many of the available studies were conducted among medical outpatient clinic attendees and community samples, with findings which cannot be generalized to patients in specific diagnostic groups like schizophrenia and other psychotic disorders [17,18]. A recent study by Mosaku & Ukpong [19] conducted among outpatients attending a psychiatric clinic in Southwest Nigeria reported a prevalence of 86.5% for erectile dysfunction, with varying prevalences reported for other forms of sexual dysfunctions. However, aside patients with schizophrenia, the study included patients diagnosed with other mental illnesses such as depression, bipolar affective disorder, and psychoactive substance dependence, who may actually have differing patterns and prevalences of sexual dysfunction. To the best knowledge of the authors, no Nigerian studies have reported the prevalence and correlates of sexual dysfunction among patients in a specific diagnostic group like those with schizophrenia and delusional disorders.

The aim of this study was to determine the prevalence of sexual dysfunction, associated socio-demographic and clinical factors among male patients with psychotic disorders on conventional antipsychotic, in Southwestern Nigeria.

## Methods

### Study design and setting

This was a descriptive cross sectional survey of sexual dysfunction among male out-patients with schizophrenia on conventional antipsychotic medications for at least six months. The study was carried out at the out-patient clinic of the Neuropsychiatric Hospital Aro, Abeokuta, Nigeria between August 2005 and February 2006. The annual report of the medical records department of the hospital showed that 3,270 patients with a diagnosis of schizophrenia attended the outpatient clinic in 2004 (Unpublished data, medical records department). The hospital has a total capacity of 526 beds and attends to all patients that come to the hospital through referrals and those brought by relatives. It has an Emergency/Assessment Unit that provides a 24-hour first-contact and Emergency services, 7 days of the week while outpatient clinics are run for follow-up consultations on Mondays, Tuesdays, Thursdays and Fridays after the first contact or following discharge from the in-patient care.

### Participants

Participants were consecutive male outpatient between the ages of 18–60 years. Only subjects meeting the ICD-10 criteria for schizophrenia and delusional disorders (F20 – F29) based on information from patients` case notes’ were included in the study. Patients with clinical history/record of conditions and medications that may contribute to sexual dysfunctions viz Diabetes, hypertension, cerebrovascular disorder e.g. stroke, gonadal injury, endocrine disorder/medications, alcohol dependence, antidepressant medication were excluded from the study (all patients undergo routine laboratory screening including fasting/random blood sugar and full physical examination at presentation and regular intervals for any concomitant physical illness at the study center; body weight and BP checks are also carried out at every visit).

### Instruments

The following instruments were administered:

1. A questionnaire drawn up by the researchers (AKO and TOA) to elicit information on socio-demographic characteristics of respondents and their clinical characteristics, such as illness and medication history.

2. Brief Psychiatric Rating Scale (BPRS) - The BPRS is a widely used instrument developed by Overall & Gorham to measure psychotic symptoms and psychopathology profiles [20]. It is a semi-structured interview schedule originally comprising 16 items. Each item is scored on a 7-point scale and produces sub scores (profiles) for affective, psychotic and negative symptoms. The 16 items of the original scale was used in this study to measure current psychopathological profile of the subjects. The BPRS has been used by previous researchers in Nigeria [21]

3. The International Index of Erectile Function (IIEF) questionnaire - This is a self administered questionnaire that evaluates male sexual functions. The IIEF was developed by an International panel of experts through an extensive review of the literature and existing questionnaires in addition to detailed interview of men with sexual dysfunction and their partners [22]. The IIEF instrument consists of 15 questions (Q), rated on a scale of 1–5, with 0 indicating no sexual activity or no attempt. It has 5 domains: Erectile dysfunction (Q1 – 5, 15), Orgasmic Function (Q9, 10), Sexual Desire (Q11, 12), Intercourse Satisfaction (Q6 – 8), and Overall Satisfaction (Q13, 14), each addressing a unique dimension of sexual function. Total IIEF questionnaire score ranged from 0–30, with higher scores indicating better sexual functioning. Responses to each question are based on a man’s experience over the past 4 weeks. The IIEF has been used by previous authors in Nigeria [19] and in their study, a reliability coefficient (cronbach’s alpha) of 0.921 was obtained.

All the questionnaires were translated to Yoruba (the predominant language in the locality of the study) through the process of back translation.

### Procedure

Consecutive male outpatient clinic attenders between the ages of 18–60 who were married and/or who had a regular sexual partner and who had fulfilled the ICD-10 criteria for schizophrenia, and delusional disorders (F20 – F29) at one time or the other based on information from patients` case notes’, and were currently on conventional antipsychotic medications for at least six months (including those that still had active or residual symptoms but not acutely disturbed with gross excitement or disorientation) were included. The interviews were conducted by AKO and two trained research assistants (resident doctors in psychiatry) in the outpatient clinic consultation rooms after routine consultation, to ensure confidentiality. Assistance in completing the questionnaires was provided for the respondents where necessary.

### Ethical considerations

Approval of the Research Ethical Committee of the Neuropsychiatric Hospital, Aro, Abeokuta, Ogun State, Nigeria was obtained to carry out the study. This study complied with the Declaration of Helsinki protocol and informed verbal consent was obtained from the participants after a detailed explanation of the study.

### Data analysis

Statistical Package for Social Sciences (SPSS) version 11.0 for Windows™ was used for data analysis. Most of the variables were grouped for ease of statistical analysis. Results were calculated as frequency (%) and mean. Group differences were determined using Chi-square (*χ*^2^) test for categorical variables and student *t*-test for continuous variables. Variables that were found to be significantly associated with any form of sexual dysfunction (independent variables) were then included in a logistic regression model with presence or absence of sexual dysfunction as the outcome (dependent variable). Level of significance was set at *p* < 0.05.

## Results

### Sociodemographic characteristics

Two hundred and seventy nine male outpatients who met the inclusion criteria were invited to participate in the study. There were 5 outright refusals, giving a response rate of 98.6%. The data of 275 male outpatients meeting the inclusion criteria for the study were analysed. Mean age was 39.5 ± 9.4 years, and they were mainly between 30 and 39 years old (36.4%). Respondents were predominantly married (60.7%) and the majority of patients (86.9%) were employed. The patients were predominantly Christians (62.9%) Table 1*.*

**Table 1:** Demographic, Clinical and Medication Related Characteristics of Respondents

**Variable**		**Frequency (%)**
Age	18–29 years	44 (16.0)
	30–39 years	100 (36.4)
	40–49 years	85 (30.9)
	50–60 years	46 (16.7)
	Mean (SD) years: 39.5 (9.4)	
Education	No Formal Education	10 (3.7)
	Primary School	84 (30.5)
	Secondary School	110 (40)
	Tertiary	71 (25.8)
Marital Status	Single	93 (33.8)
	Married	167 (60.7)
	Separated	4 (1.5)
	Divorced	8 (2.9)
	Widowed	3 (1.1)
Employment Status	Employed	239 (86.9)
	Unemployed	36 (13.1)
Religion	Christian	173 (62.9)
	Muslim	101 (36.7)
	Traditional Worshipper	1 (0.4)
Age at onset of illness	<15 yrs	6 (2.2)
	15–25 yrs	117 (42.5)
	26–35 yrs	111 (40.4)
	>35	41 (14.9)
	Mean (SD): 27.4 (7.4)	
Diagnosis	Schizophrenia	241 (87.7)
	Persistent delusional disorder	1 (0.4)
	Acute & transient psychotic disorder	2 (0.7)
	Schizoaffective disorder	2 (0.7)
	Unspecified non organic psychotic disorder	29 (10.5)
Duration of Antipsychotic Medication Use	<2 yrs	28 (10.2)
	2–5 yrs	56 (20.4)
	5–10 yrs	60 (21.8)
	>10	131 (47.6)
	Mean (SD): 8.4 (2.1)	
Daily medication Dose*	<500 mg	170 (61.8)
	500–1000 mg	82 (29.8)
	>1000mgs	23 (8.4)
	Mean: 462 mg	
Number of Antipsychotics	One	97 (35.3)
	Two	152 (55.3)
	Three or more	26 (9.4)

*In Chlorpromazine equivalents.

### Clinical characteristics and medication related variables

The majority of patients (87.6%) had a diagnosis of schizophrenia. The mean age at onset of illness was 27.4 ± 7.4 years. The majority of respondents (42.5%) had their onset of illness between 15 and 25 years. The mean (SD) BPRS score was 0.40 (1.2).

The mean duration of conventional antipsychotic medication use was 8.4 ± 2.1 years. One hundred and thirty one patients (47.6%) had been using medications for more than 35 years. Majority of the patients (55.3%) were taking more than two conventional antipsychotic at the time of the study. The mean chlorpromazine equivalent daily medication dose taken by the patients was 462 mg, with the majority (61.8%) being maintained on less than 500 mg chlorpromazine equivalent daily dose (Table 1).

### Prevalence of sexual dysfunction

One or more forms of sexual dysfunction existed among 111 (40.4%) of the respondents. Sexual desire dysfunction was present in 47 (17.1%) of subjects, Erectile dysfunction in 95 (34.5%), Orgasmic Dysfunctions 51 (18.5%), Intercourse Dissatisfaction 72 (26.2%) and Overall Dissatisfaction 64 (23.3%).

### Correlates of sexual dysfunction

The demographic, medication and illness related variables associated with one or more forms of sexual dysfunction were: Employment status (sexual desire dysfunction, orgasmic dysfunction, intercourse dissatisfaction and overall dissatisfaction), age group (orgasmic dysfunction), marital status (overall dissatisfaction), haloperidol use (erectile dysfunction, orgasmic dysfunction), medication dose (erectile dysfunction, orgasmic dysfunction, overall dissatisfaction), any psychopathology on the BPRS (overall dissatisfaction) Table 2.

**Table 2:** Association between Specific Sexual dysfunctions and Sociodemographic, Illness-related and Medication-related Variables

**VARIABLES OPTIONS**		**Sexual Desire**	**Erectile**	**Orgasmic**	**Intercourse**	**Overall**
		**Dysfunction**	**Dysfunction**	**Dysfunction**	**Dissatisfaction**	**Dissatisfaction**
		***χ***^**2**^**,*df,*(p -Value)**	***χ***^**2**^**,*df,*(p -Value)**	***χ***^**2**^**,*df,*(p -Value)**	***χ***^**2**^**,*df,*(p -Value)**	***χ***^**2**^**,*df,*(p -Value)**
**Sociodemographic**						
Age Group	<30 yrs/31–40 yrs/41–50 yrs/51–60 yrs	4.330, 3, (0.228)	4.581, 3, (0.205)	9.231, 3, (**0.026**)	5.111,3, (0.164)	3.514, 3, (0.319)
Age at Onset of Illness	<15 yrs/15–25 yrs/26–35 yrs/>35 yrs	1.997, 3, (0.573)	1.329, 3, (0.722)	2.635, 3, (0.451)	1.435, 3, (0.697)	0.688, 3, (0.876)
Marital Status	Married/Single/Separated/Divorced/Widowed	1.653,4, (0.799)	5.332, 4, (0.255)	6.084, 4, (0.193)	8.543, 4, (0.074)	12.067, 4,(**0.017**)
Level of Education	Nil/Primary/Secondary/Tertiary	5.659, 3, (0.129)	1.709, 3, (0.635)	2.718, 3, (0.437)	5.281,3, (0.152)	0.106, 3, (0.991)
Employment Status	Employed/Unemployed	9.087, 1, (**0.003**)	2.334, 1, (0.092)	9.852, 1, (**0.002**)	4.259, 1, (**0.039**)	4.696, 1, (**0.030**)
**Medication Related**						
Duration of Medication Use	<2 yrs/2–5 yrs/6–10 yrs/>10 yrs	1.013, 3, (0.798)	1.566, 3, (0.667)	2.343, 3, (0.504)	1.794, 3, (0.616)	6.215, 3, (0.102)
Number of Medications	One/Two/Three or more	3.537, 2, (0.171)	4.506, 2, (0.105)	1.960, 2, (0.375)	0.687, 2, (0.709)	3.390, 2, (0.184)
Haloperidol	Yes/No	0.697, 1, (0.404)	4.979, 1, (**0.026**)	4.620, 1, (**0.032**)	1.041, 1, (0.308)	3.145, 1, (0.076)
Chlorpromazine	Yes/No	0.041, 1, (0.713)	0.053, 1, (0.818)	0.136, 1, (0.712)	0.005, 1, (0.943)	0.000, 1, (1.000)
Thioridazine	Yes/No	0.000, 1, (1.000)	0.547, 1, (0.460)	0.618, 1, (0.432)	0.084, 1, (0.772)	0.014, 1, (0.907)
Trifluoperazine	Yes/No	0.000, 1, (1.000)	0.000, 1, (1.000)	0.116, 1, (0.733)	0.003, 1, (0.956)	0.036, 1, (0.850)
Fluphenazinedecanoate	Yes/No	1.164, 1, (0.281)	1.357, 1, (0.244)	0.000, 1, (1.000)	0.007, 1, (0.933)	0.815, 1, (0.367)
Flupenthixoldecanoate	Yes/No	0.181, 1, (0.671)	0.000, 1, (1.000)	0.246, 1, (0.620)	0.000, 1, (1.000)	0.000, 1, (1.000)
Dose of Medication^*^	<500 mg/500 mg-1000 mg/>1000 mg	5.179, 2, (0.075)	6.851, 2, (**0.033**)	7.094, 2, (**0.029**)	4.197, 2, (0.123)	6.929, 2, (**0.031**)
**Illness Related**						
Diagnosis^†^	Schiz/PDD/APD/SZA/UPD	2.149, 4, (0.708)	4.769, 4, (0.312)	3.015, 4, (0.555)	6.669, 4, (0.154)	1.943, 4, (0.746)
Any Psychopathology on BPRS	Present/Not Present	2.293, 1, (0.130)	0.031, 1, (0.860)	0.075, 1, (0.784)	0.000, 1, (1.000)	5.056, 1, (**0.025**)

^*^Chlorpromazine Equivalents;

^†^ Schiz – Schizophrenia; PDD – Persistent Delusional Disorder; APD – Acute Psychotic Disorder; SZA – Schizoaffective Disorder; UPD – Unspecified Psychotic Disorder.

BPRS – Brief Psychiatric Rating Scale.

Variables having significant association with any form of sexual dysfunction are in bold.

### Independent correlates of sexual dysfunction

Result of the logistic regression analysis showed unemployment as the only independent correlate of sexual dysfunction, with unemployed respondents twice as likely to have sexual dysfunction as those employed (Wald = 3.865, Odds ratio = 2.033, 95% confidence interval = 1.002 - 4.124, p = 0.049).

## Discussion

This study examined the prevalence and correlates of sexual dysfunction among Nigerian men with psychotic illness attending a psychiatric outpatient clinic.

Overall, about 40% of the respondents had at least one form of sexual dysfunction. This rate of sexual dysfunction is similar to that reported in other previous studies [8,10,18]. Considering the finding that the mean age (39.5 years) of the respondents fell within the reproductive age group, problems with their sexual functioning may be a significant source of concern for them with far reaching consequences if left untreated.

Most of the patients (64.7%) in this study were taking more than one conventional antipsychotics, an observation also made in a study examining the prescribing habits for psychiatric in-patient admissions [23]. Other authors have found that despite extensive research and recommendations regarding the rational prescription of antipsychotic drugs, polypharmacy exists even among clinically stable patients [24].

This study reports an association between poly-pharmacy and sexual dysfunction which can be explained by the fact that increasing the number of medications result in increased risk of adverse effects experienced by patients. Our finding is similar to what previous authors reported that combination of antipsychotics adds to the risks of developing medication side effects [25]. In addition, we found a significant relationship between medication dosages (chlorpromazine equivalents) and some forms of sexual dysfunction, similar to that reported by some authors [7]. Higher dosages of conventional antipsychotics are therefore associated with higher prevalence of sexual dysfunction since there will be more drugs to act at the various pathways leading to sexual dysfunction [8].

Among the conventional antipsychotics medications prescribed, only haloperidol showed a significant relationship with sexual dysfunction (erectile and orgasmic dysfunctions). This may be attributed to the high affinity of haloperidol for dopamine D_2_ receptor and inhibition of dopamine release, resulting in impaired libido and erection [26]. On the other hand, the sexual dysfunction observed may be a result of severe psychopathologies experienced by the patients [4], which then necessitated the use of a highly potent typical antipsychotic medication like haloperidol.

Erectile dysfunction was the commonest type of sexual dysfunction reported by the respondents, a finding also reported in previous studies [19,27,28]. Poor penile erection interferes with subjective enjoyment of other stages of sexual intercourse and because it was the commonest sexual dysfunction reported by respondents, it may account for the high prevalence of intercourse dissatisfaction and overall dissatisfaction with sex observed in this sample. Inability to achieve good penile erection for optimal sexual satisfaction may be associated with feelings of inadequacy in the sufferer. In many societies, including Nigeria, individuals with poor or absent penile erection are often stigmatized, subjected to public ridicule and may be deserted by their spouse. Erectile dysfunction may result in poor treatment adherence and negatively impacts on patients’ quality of life [15].

Our study revealed a significant relationship between marital status and sexual dysfunction, specifically with overall dissatisfaction with sex. It may be that patients with medication induced sexual dysfunction were likely to be dissatisfied with their overall sexual functioning. For married men, the marriage setting provides an opportunity for a ‘feedback’ from the spouse about sexual performance, which may result in subjective awareness of an existing sexual inadequacy, otherwise unnoticed by the patient. This raises a possibility that the relationship between marital status and sexual dysfunction seen in these patients may be psychogenic in origin, rather than organic. Unfortunately, the IEFF could not distinguish between organic and psychogenic sexual dysfunction, an important limitation of this study. However, other authors have also reported a significant association between marital status and sexual dysfunction among patients taking conventional antipsychotics in Nigeria [19]

In this study, unemployed respondents were more likely to have sexual dysfunction than those who are employed. Previous studies have found an association between sexual dysfunction, depression and socioeconomic disadvantages like unemployment [8,29]. Unemployment may result in role reversal within a relationship, engendering feelings of shame and inadequacy in the male partner. Previous authors have reported that unemployment in a person with mental illness is associated with public and self-stigmatization, a “double jeopardy”, which may negatively impact on self-worth and sexual performance or satisfaction [21].

This study has a number of limitations. First, it was cross-sectional in nature, so the direction of causality between sexual dysfunction and the sociodemographic and clinical variables could not be inferred from the findings. Second, there is a limitation regarding the generalizability of the result to other patients on conventional antipsychotics in Nigeria, as the study was conducted in just one centre. Third, the absence of a control group is also an important limitation to the generalizability of our results. However, to the best of our knowledge, it is the first to examine sexual dysfunction among specific group of psychiatric outpatients on conventional antipsychotics in Nigeria. It is also one of the few available studies on sexual dysfunction in a developing country setting where conventional antipsychotic medications are commonly prescribed for the treatment of psychotic illnesses [30].

## Conclusions

Sexual dysfunction is common among outpatients with psychotic disorders on conventional antipsychotics. It is associated with demographic, illness and medication related variables. Unemployment was found to be the most important independent correlate of sexual dysfunction. Therefore, there is a need among clinicians for increased awareness and recognition of the sexual side effects of conventional medications on patients, especially those socially disadvantaged. This should guide antipsychotic medication prescription resulting possible improvement in treatment adherence and outcome.

## Competing interests

The authors declare that they have no competing interests.

## Authors’ contribution

OAK conceived the study and together with ATO designed the study. OAK executed the data collection. AOA and OA did the statistical analysis and drafted the first version of the manuscript. OAK and ATO participated in the interpretation of data. All authors read and approved the final manuscript.

## Source of financial support

None.
